# Metastatic cardiac tumors: from clinical presentation through diagnosis to treatment

**DOI:** 10.1186/s12885-018-4070-x

**Published:** 2018-02-20

**Authors:** Ivana Burazor, Sarit Aviel-Ronen, Massimo Imazio, Orly Goitein, Marina Perelman, Natalia Shelestovich, Ninoslav Radovanovic, Vladimir Kanjuh, Iris Barshack, Yehuda Adler

**Affiliations:** 1 Department of Cardiology, Institute for Rehabilitation, Belgrade, Serbia; 2 Medical Faculty, University of Belgrade, Belgrade, Serbia; 3 Department of Pathology, Sheba Medical Center, Ramat-Gan, Israel; 4 Talpiot Medical Leadership Program, Sheba Medical Center, Ramat-Gan, Israel; 5 Department of Cardiology, Maria Vittoria Hospital, Torino, Italy; 6 Department of Diagnostic Imaging, Sheba Medical Center, Ramat-Gan, Israel; 7 Sackler Faculty of Medicine, Tel Aviv University, Tel Aviv, Israel; 8 Institute for Cardiovascular Diseases of Vojvodina, Sremska Kamenica, Serbia; 9 The Board on Cardiovascular Pathology, Department of Medical Sciences, Serbian Academy of Sciences and Arts, Belgrade, Serbia

**Keywords:** Metastatic cardiac tumors, Myocardium, Melanoma, Metastatic carcinoma, Metastatic sarcoma

## Abstract

**Background:**

To evaluate the prevalence of metastatic tumors involving the myocardium and study their presentation in order to increase awareness to their existence.

**Methods:**

Pathological reports from Sheba Medical Center (Israel, January 1, 2010 through December 31, 2015) and medical records from The Institute for Cardiovascular Diseases of Vojvodina, Sremska Kamenica (Serbia, 23 years period) were screened for cases of metastatic cardiac tumors. Medical, radiological and pathological data of identified cases was retrieved and reviewed.

**Results:**

Out of thousands of registered cardiac surgeries we found less than a dozen cases of metastatic cardiac tumors classified as melanoma, carcinomas of lung, colon and kidney and sarcomas of uterine origin. We found that metastatic cardiac tumors comprised 15.8% of all the cardiac tumors.

**Conclusions:**

Metastatic cardiac tumors are extremely rare. As new diagnostic technologies and improved survival of oncological patients may increase the incidence of metastatic cardiac tumors in the future, awareness to their existence and knowledge of their presentation are key factors in their timely recognition.

## Background

Metastatic cardiac tumors (MCTs) involving the myocardium and pericardium are rare, with reported incidence of 1.5–20% of autopsies of cancer patients [1]. Though these metastases may originate from any malignant tumor, they most commonly are caused by melanoma, lymphoma, leukemia and carcinoma of the lung, breast and esophagus. Most cardiac secondary tumors remain clinically silent (over 90%) and are often diagnosed postmortem [1–3].

In view of the scarcity of these tumors and with the attempt to raise awareness to their existence, it was our aim to study the prevalence of MCTs in our institutes and analyze their presentation. We present our experience with MCTs in two medical centers and highlight several rare cases. We review the potential differential diagnosis, the diagnostic tools utilized to identify these metastases, the pathological mechanisms for tumor spread into the heart, the available treatment modalities, and the prognosis of cardiac metastases. Finally, we present a review of the literature.

## Methods

We collected data from pathology reports at Sheba Medical Center (SMC), Tel Hashomer, Israel and from medical records at the cardiosurgery clinics at The Institute for Cardiovascular Diseases of Vojvodina (TICDV), Sremska Kamenica, Serbia. At SMC, the search for MCTs from the electronic pathological reports was based on SNOMED (Systematized Nomenclature of Medicine) code routinely given to each case. Data was available from January 1, 2010 through December 31, 2015 during which 155,205 non-cardiac and cardiac cases were received for pathological evaluation at the department of pathology SMC. In the Serbian center, data was retrieved from cardiac surgical cases reported in the medical records and the electronic database. Data was available for 23 years period during which 20,998 cardiac surgeries were performed at TICDV. Radiological and pathological information was available only for the cases from SMC. The study was approved by the SMC Institutional Helsinki Committee (approval no. 3235–16- SMC, valid till June 16, 2017) and conforms with Serbian and TICDV regulations for use of clinical data and research.

## Results

Only a handful of MCTs were identified in both medical centers. In SMC, we diagnosed 3 cases of MCTs (melanoma, colonic and lung carcinomas) from 2010 to 2015, out of 155,205 non-cardiac and cardiac biopsies and 55 adult autopsies (diagnosed in 1.8% of autopsies). Throughout the study period, 12 benign cardiac tumors (8 myxomas, 4 papillary fibroelastomas) and 4 malignant primary cardiac tumors (2 high grade sarcomas and 2 high grade lymphomas) were diagnosed. Thus, metastatic tumors comprised 15.8% of all the cardiac tumors.

In TICDV, out of 20,998 open heart surgeries performed during 23 years period, only 5 patients had cardiac manifestation of extra-cardiac tumors (2 uterine sarcomas, 2 lung carcinomas and one renal carcinoma).

The age of patients with MCTs ranged from 39 to 77 years with just over half in their sixth and seventh decade of life. Six patients were women and 2 were men (Table 1). Following is a detailed description of the SMC cases.

**Table 1:** Metastatic cardiac tumors – our experience from two separate centers

Department	Pathology	Cardiosurgery
Center	Sheba Medical Center	Institute for Cardiovascular Diseases of Vojvodina
Period (years)	5 years	23 years
Total number of cases	155,205	20,988
Metastatic tumors	3	5
Case details	1. Metastatic melanoma in the intracavitary region of the right atrium- 5th decade patient 2. Metastatic colonic poorly differentiated adenocarcinoma in the right atrium – 7th decade patient 3. Metastatic lung basaloid squamous cell carcinoma in the septum and apex of the heart – 8th decade patient	1. Metastatic uterine leiomyosarcoma in the right atrium - 5th decade patient 2. Metastatic lung squamous cell carcinoma in the left atrium - 7th decade patient 3. Metastatic clear cell renal cell carcinoma in the left atrium- 6th decade patient 4. Metastatic lung adenocarcinoma in the left atrium - 7th decade patient 5. Metastatic uterine leiomysarcoma - 4th decade patient

### *Case Number 1.* Metastatic melanoma in the intracavitary region of the right atrium

**Fig. 1:**
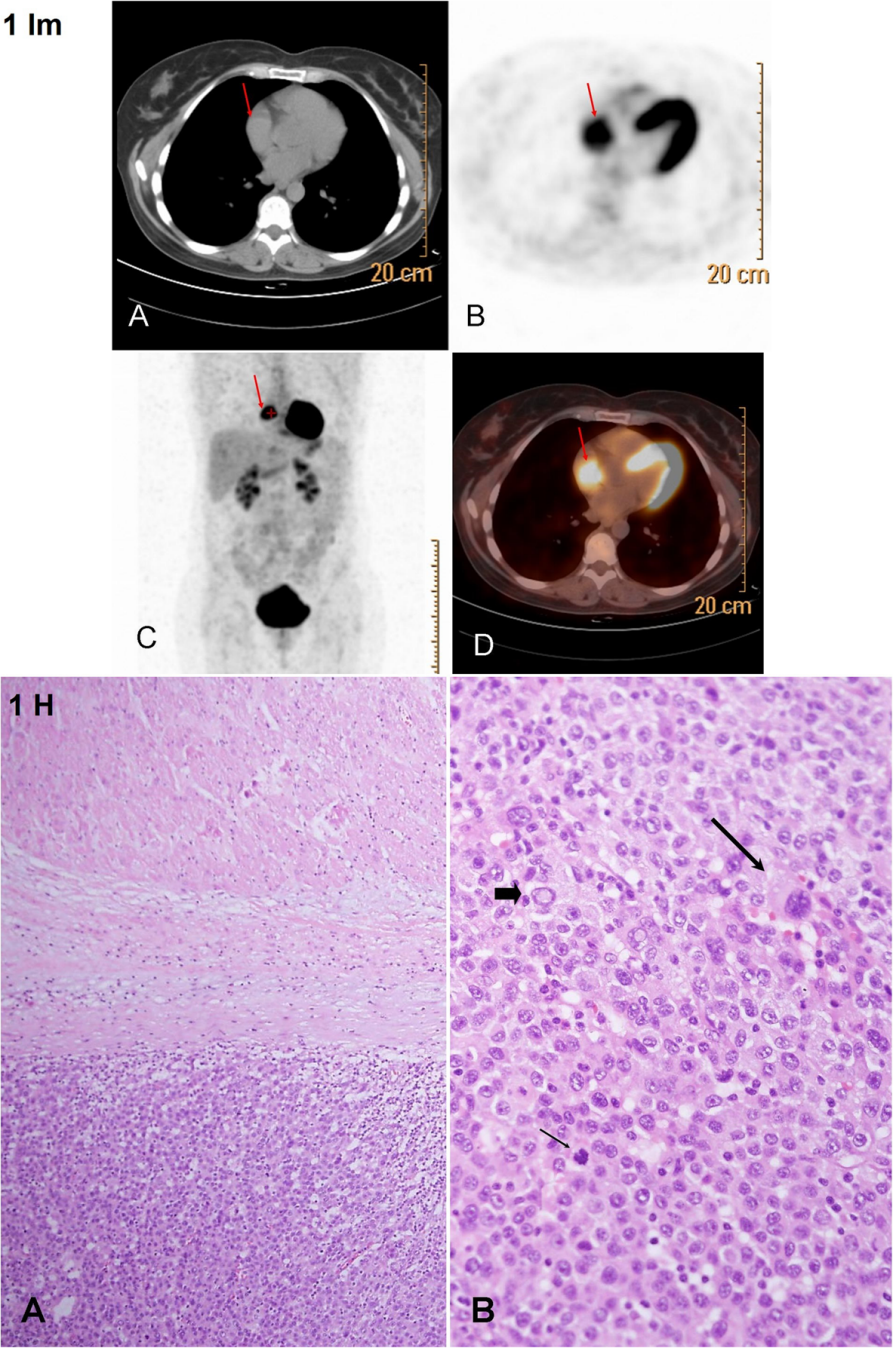
Im: Case no. 1: Metastatic melanoma in the right atrium - Imaging. **a** – Axial non contrast CT section (part of the positron emission tomography CT): no obvious mass is demonstrated in the RA (red arrow) due to the lack of contrast administration. **b** – positron emission tomography CT, axial section demonstrating the right atrium mass with significant fluorodeoxyglucose uptake (red arrow), consistent with high metabolic activity. **c** – Whole body positron emission tomography CT, coronal section, the right atrium mass depicts substantial fluorodeoxyglucose uptake (red arrow). **d** – Fused positron emission tomography CT image, axial section (parallel to **a** & **b**) showing the right atrium mass along with the fluorodeoxyglucose uptake (red arrow). Fig. 1 H: Case no. 1: Metastatic melanoma in the right atrium - Histology. **a** - Cardiac muscle is seen in the upper part, separated by fibrosis and mild inflammatory infiltrate from a metastatic melanoma mass seen in the lower part, HE X100. **b** – Metastatic melanoma growing in solid sheets of cells. The melanoma cells show typical features of abundant cytoplasm, large nuclei with prominent nucleoli, occasional intranuclear inclusions (wide short arrow), bizarre atypical cells (thin long arrow) and numerous mitotic figures (thin short arrow), HE X400

A patient in the 5th decade with history of metastatic malignant melanoma performed a periodic follow-up positron emission tomography CT. During the 7 years since the diagnosis, the patient experienced 4 recurrences of metastatic tumors that were all resected and followed by various modes of immunotherapy (interferon-alpha and tumor infiltrating lymphocytes). A scheduled positron emission tomography CT demonstrated a mass in the right atrium (Fig. 1 Im). No other metastatic lesions were identified. Echocardiography showed right atrium mass. There was normal function of the ventricles and valves and no evidence of a pericardial effusion. Transesophageal echocardiogram verified that the atrial mass was pedunculated and surgical intervention proceeded. A mass measuring 3.5 × 4 × 4.5 cm was resected from the right atrium. The mass presented histomorphological features of malignant melanoma and was attached to the myocardial wall (Fig. 1 H, large lesion, representative samples were examined.). A year later, a single brain metastasis was removed by radiosurgery and the following year the patient commenced a long-term B-Raf inhibitor treatment with Vemurafenib (Zelboraf). After five years of follow up the patient is alive with no symptoms.

### *Case Number 2*: Poorly differentiated adenocarcinoma of the colon, metastatic to the right atrium

**Fig. 2:**
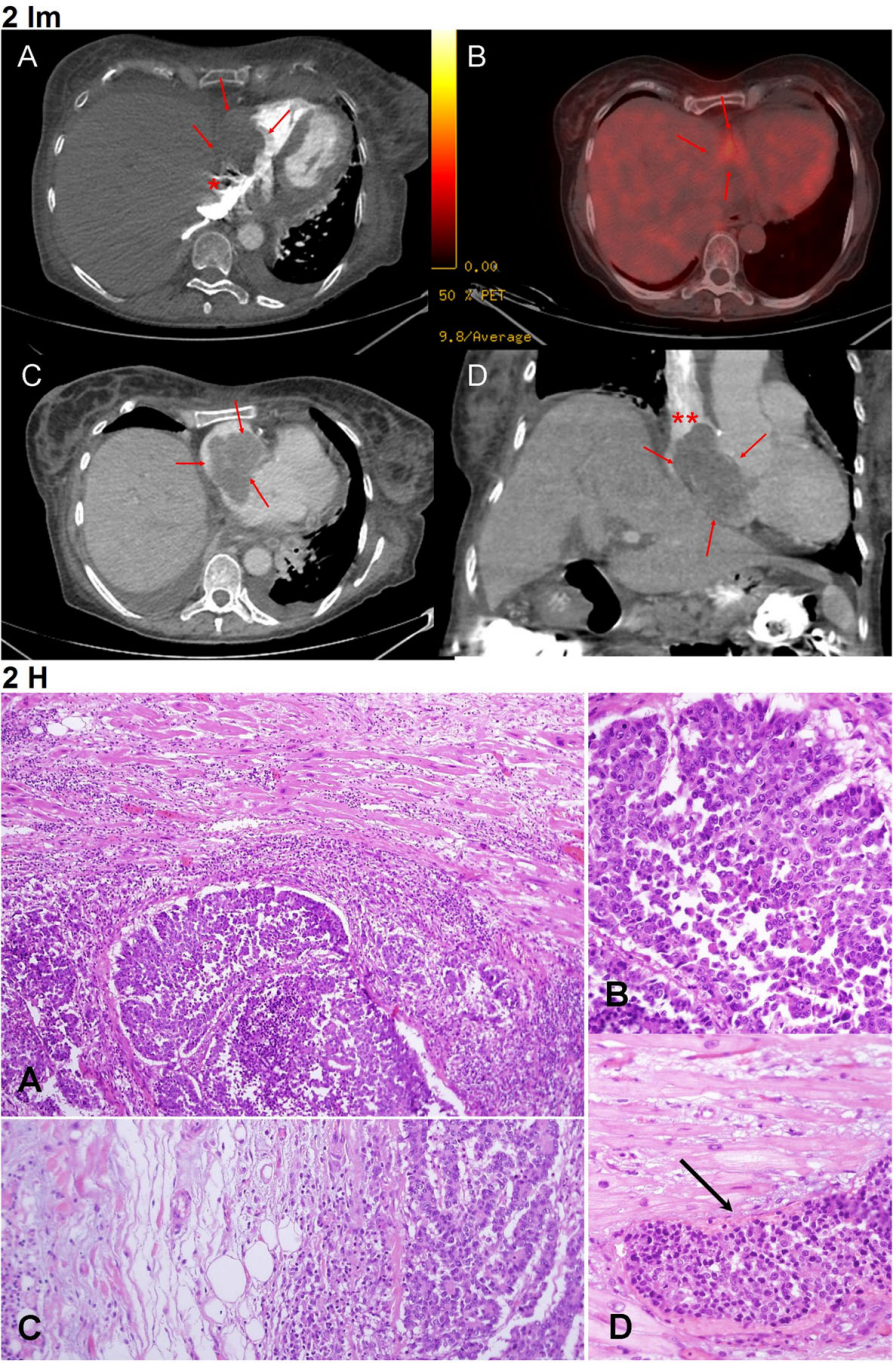
Im: Case no.2: Poorly differentiated adenocarcinoma of the colon, metastatic to the right atrium - Imaging **a** - Contrast enhanced CT axial section demonstrating a large right atrium mass (red arrows). The mass protrudes into the IVC (asterisks). **b** – Fused positron emission tomography CT image, axial section, the right atrium mass is highlighted by fluorodeoxyglucose uptake consistent with metabolic activity (red arrow). **c** – Contrast enhanced CT axial section demonstrating a non-enhancing right atrium mass filling the entire right atrium (red arrows). **d** – Contrast enhanced CT coronal section showing the large right atrium mass (red arrows) protruding into the superior vena cava (red asterisks). Fig. 2 H: Case no.2: Poorly differentiated adenocarcinoma of the colon, metastatic to the right atrium - Histology. **a** - Cardiac muscle is seen in the upper part, infiltrate by carcinoma growing in solid to vaguely glandular pattern, seen in the lower part. The cardiac cells show enlarged reactive nuclei and are surrounded by inflammatory infiltrate, HE X100. **b** – Poorly differentiated carcinoma, HE X400. **c** – Carcinoma invading into the pericardial adipose tissue, HE X 200. **d** – Vascular invasion of carcinoma into a small cardiac blood vessel (thin long arrow), HE X400

A patient in the 7th decade was admitted with clinical presentation of superior vena cava syndrome. During the two weeks prior to the admission the patient developed a cough, dyspnea, facial edema and deterioration in functional ability. Positron emission tomography CT (Fig. 2 Im) demonstrated a hypermetabolic mass in the right atrium protruding into the superior vena cava. Eleven months before, a right adrenalectomy and right hemicolectomy were performed due to poorly-differentiated adenocarcinoma. Although surgical resection of the cardiac mass was considered, the patient’s general condition did not allow this intervention. The patient died after a week of hospitalization, during which hemodynamic instability necessitated mechanical ventilation. On the autopsy performed, a mass measuring 6 × 6 × 3 cm was found attached to the right atrium wall, morphologically compatible with poorly differentiated adenocarcinoma (Fig. 2 H, large lesion, representative samples were examined.). Additional findings included disseminated metastatic adenocarcinoma involving the lung, left adrenal, liver, thyroid and numerous lymph nodes.

### Case number 3: Metastatic lung basaloid squamous cell carcinoma in the septum and apex of the heart

**Fig. 3:**
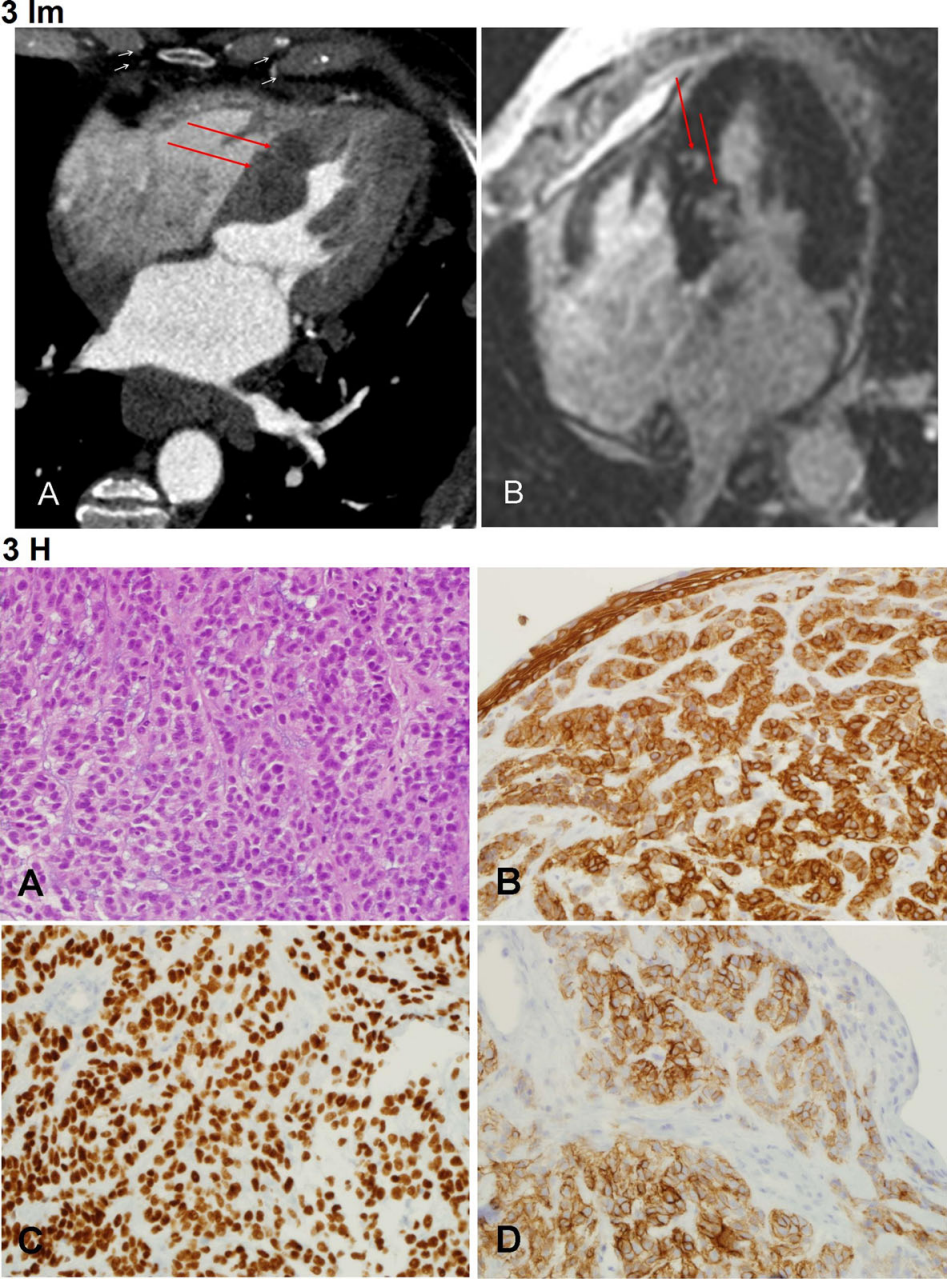
Im: Case no. 3: Metastatic lung basaloid squamous cell carcinoma in the septum and apex of the heart - Imaging. **a** - Cardiac CT: Four chamber view demonstrating hypodense round masses in the interventicular septum (red arrows). **b** - Cardiac MRI: Four chamber view, delayed enhancement; (normal muscle is hypo-intense - black), two enhancing foci (white) are demonstrated in the interventricular septum (red arrows). Fig. 3 H: Case no. 3: Metastatic lung basaloid squamous cell carcinoma in the septum and apex of the heart - Histology. This lung carcinoma is composed of monomorphic cells with moderate amount of cytoplasm and small prominent nucleoli growing in a trabecular-like pattern. Positive immunosatining for CK 5/6 and p63 demonstrate the basaloid features and enhance the trabecular-like growth pattern. Positive immunostaining for CD56 highlights some neuroendofrine features. **a** - HE X400. **b** – Immunosatin for CK5/6 X400. **c** - Immunosatin for p63 X400. **d** - Immunosatin for CD56 X400

A patient in the 8th decade presented with hemoptysis. Echocardiography showed ejection fraction of 65%, hypertrophic septum with no metastatic involvement and a small pericardial effusion. Moderate aortic stenosis with moderate aortic regurgitation were also detected. A CT scan demonstrated a lung mass in the left hilum and a cardiac CT and cardiac magnetic resonance imaging (MRI) demonstrated involvement of the interventricular septum (Fig. 3 Im). The lung mass biopsy showed a basaloid squamous cell carcinoma, positive for the neuroendocrine immuno-marker CD56 (Fig. 3 H, endobronchial biopsy - all material was submitted for histologic examination). The patient’s clinical presentation was dominated by his primary lung malignancy and cardiac surgery was not indicated. The patient died 9 months after the lung cancer diagnosis, due to his primary malignancy.

The next case exemplifies the clinical mimickers of MCTs and the differential diagnosis.

### Case no. 4: Metastatic testicular germ cells tumor in the liver with giant mass in the right atrium.

**Fig. 4:**
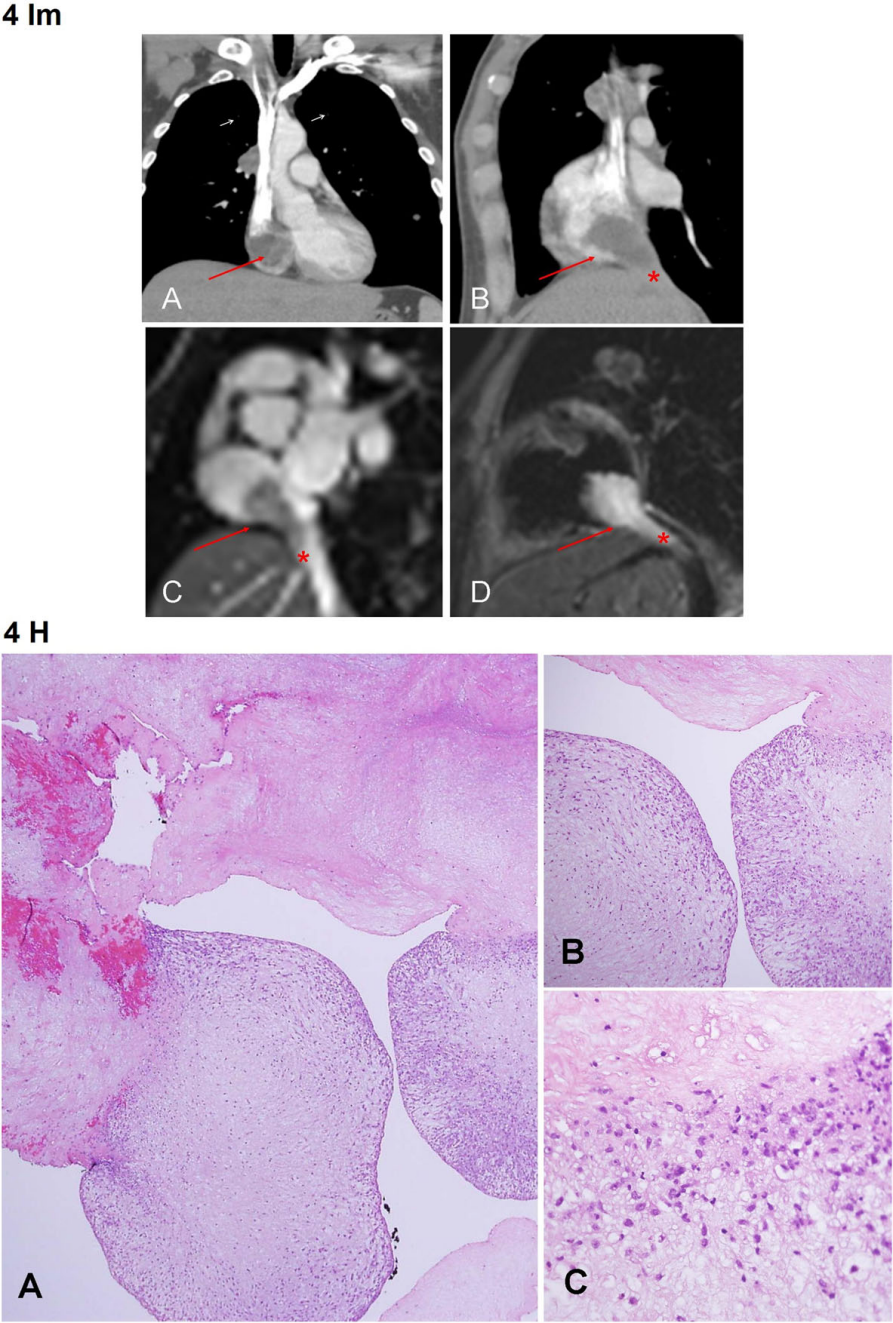
Im: Case no. 4: Metastatic testicular germ cells tumor in the liver with thrombus of the right atrium - Imaging. **a** – Contrast enhanced CT coronal reconstruction showing a hypodense right atrium mass (arrow). **b** - Contrast enhanced CT sagittal reconstruction demonstrating the hypodense right atrium mass (arrow) continuing into the IVC (asterisk). **c** – Cardiac MRI steady stated free precession sequence (“bright blood”): a plane through the right atrium and IVC. A hypointense mass is demonstrated in the right atrium (arrow) protruding into IVC (asterisk). **d** - Cardiac MRI T2 sequence: a plane through the right atrium and IVC- a hyperintense mass is demonstrated in the RA (arrow) protruding into IVC (asterisk). Fig. 4 H: Case no. 4: Metastatic testicular germ cells tumor in the liver with giant thrombus of the right atrium - Histology. **a** Most of the cardiac mass consisted of amorphic eosinophilic material, seen in the upper part, representing fibrin of a thrombus. Foci of initial thrombus organization are seen at the bottom, HE X40. In the areas of initial thrombus organization **b)** and **c**) spindled mesenchymal cells are present, haphazardly arranged. Unlike in myxoma, these mesenchymal cells do not create cords, vascular channels or nests. B – HE X100, C – HE. X400. (HE = Hematoxylin and Eosin stain)

A patient in the 4th decade with a known metastatic testicular high-grade non-seminomatous germ cell tumor was admitted due to a cardiac mass (Fig. 4 Im) that was discovered during a routine follow-up CT. At first presentation, 6 years earlier, the patient had massive involvement of the liver and retroperitoneum that was treated with orchiectomy and chemotherapy and later, with retroperitoneal lymph node dissection and left nephrectomy. Following, the patient developed liver metastases that relapsed after surgical resection, and was given several courses of chemotherapy. At admission, the patient had 3 small metastases in both lungs and few metastases in the liver. In addition, a mass that involved the hepatic vein and the inferior vena cava, the latter of which extended into the right atrium. Despite these findings and all the treatments given so far, the patient remained functional with well-preserved liver, renal and pulmonary function. Echocardiography recorded a right atrium mass, a 40% ejection fraction and normal size and function of the ventricles and valves. A collaborative team of cardiac and hepatobilliary surgeons resected both the cardiac and liver masses. The liver mass was diagnosed as unclassified epithelioid malignant tumor. However, the polypoid right atrium mass that measured 2.5 × 2.5 × 4.5 cm was concluded as a thrombus (Fig. 4 Im, all the material was submitted for histologic examination). Eleven months later, the patient succumbed to his primary malignancy due to hepatic failure.

From the Serbian patient population, four died within a period of 1–16 months postoperatively. One patient with metastatic uterine leiomyosarcoma is still in the early postoperative period and is currently recovering from the operation.

## Discussion

Secondary tumors of the heart are rare [4] but are anticipated to increase with extended survival of oncologic patients thanks to improved diagnostic and therapeutic modalities.

We found that MCT comprised 15.8% of all cardiac tumors and were found in 1.8% of autopsies, similar to previously reported incidence of 15–20% [1, 5], showing increase in incidence from 1970 [1, 2, 6]. Likewise, in previous reports, we found that most MCT were diagnosed in the sixth and seventh decades of life, but in contrast to reported equal gender preference, in our cohorts more women than men had MCTs. Similar are the results recently published by Stecker et all [7]. During the 10-years-experience at the German Heart Center, MCTs were find in 9 patients (30–84 years of age, mean 58,8 years), out of 71 patients with cardiac tumors.

Every malignant tumor may metastasize to the heart, but the leading causes for cardiac metastasis are: melanoma, carcinoma (lung, breast, esophagus and rarely colorectal) and hematologic malignancies (leukemia and lymphoma) [6, 8, 9].

Malignant melanoma, is the neoplasm with the greatest propensity for cardiac involvement, as seen in 64% of patients with metastatic melanoma [10]. However, intracardiac melanoma, as presented in Case No.1, is evident in less than 2% of living patients [11].

Colorectal cancer rarely metastasizes to the heart, described in only several published case reports, with an incidence of 1.4–2% in autopsy series [12]. If cardiac metastases are present, most commonly they involve the pericardium. Metastatic involvement of the right atrium by colorectal cancer, as presented in Case No. 2, has also been described [12].

Cardiac metastasis from lung cancer is rarely diagnosed antemortem, as it usually causes no symptoms or signs [13]. In Case No. 3, which highlighted a metastatic basaloid squamous cell lung carcinoma, the clinical findings were predominantly caused by the primary lung malignancy and not by the cardiac metastases.

Metastatic heart involvement by testicular germ cells tumors has been previously reported [14]. However, the presented Case No. 4, that was clinically suspected to be metastatic disease, proved to be a thrombus.

### Tumor spread and cardiac involvement

Tumors spread can occur, in descending order, to the pericardium, myocardium (Fig. 3 Im), epicardium, endocardium and less frequently to the intracavitary regions with predominance to the right-side of the heart (Figs. 1 Im and 3). Possible pathways for cardiac tumor spread are:

1. *Hematogenous*: seen in melanoma (Case No. 1, Fig. 1 Im and 2), lymphoma and sarcoma and associated with myocardial and endocardial metastases.
2. *Lymphatic*: characteristic for lung carcinoma and results in pericardial and epicardial involvement, (Case No. 3, Fig. 3 Im and 6).
3. *Transvenous*: typical for renal cell carcinoma, hepatocellular carcinoma, leiomyoma of the uterus and pheochromocytoma that extends through inferior vena cava into the right atrium
4. *Direct*: encountered in locally aggressive tumors such as mediastinal and pleural tumors as well as carcinomas of the breast.

### Clinical presentation

The clinical manifestations of MCTs are nonspecific and depend on their location. There is no strong correlation between extent of cardiac involvement and the clinical manifestation [15]. Most secondary tumors of the heart are clinically silent (over 90%) [1], and are often diagnosed only postmortem. Therefore, early detection of these tumors is extremely challenging. At the time of initial tumor staging, metastases of the heart are usually not detected. Typically, cardiac metastases remain clinically silent until many years after cancer diagnosis. For example, cardiac metastases of malignant melanoma have been reported 14–22 years after the initial diagnosis [16, 17]. Thus, if oncologic patient presents with new onset of cardiac symptoms, clinicians should be aware of the possibility of cardiac metastases and perform further investigations [18].

MCTs may present with a wide spectrum of common cardiac symptoms and signs, but usually they present with right-sided heart failure. Symptoms attributed to arrhythmias can be seen and are due to invasion of the cardiac conduction system [19]. MCTs can also mimic cardiac ischemia due to invasion, compression or embolism of coronary arteries [20]. A life-threatening manifestation can occur when cardiac tamponade from pericardial involvement is present.

### Diagnosis and differential diagnosis

To date, there are no physical or laboratory examinations that specifically detect cardiac metastases. Nevertheless, the timely identification of patients with cardiac metastases is of outmost importance for the effectiveness of therapy. None of the signs of cardiac metastases are specific. Therefore, physical examinations can detect various murmurs and electrocardiogram can document diverse changes but imaging studies play a key role in the diagnosis.

Chest radiography may demonstrate cardiomegaly with “water bottle” sign or an enlarged azygos vein if pericardial effusion is present [21]. Yet, echocardiography remains the most efficient method for initial diagnosis. It assesses clinical signs and symptoms of heart failure, detects cardiac masses and pericardial effusion and provides information on the location, size and mobility of cardiac masses.

Supplemental diagnostic imaging methods are computed tomography, positron emission tomography CT and magnetic resonance tomography that add information on the tumor size and morphology, location, invasiveness, and vascularization. These methods provide sections of cardiac, mediastinal, pulmonary and thoracic structures in any plan without overlapping. Administration of contrast may be of help, with F-fluorodeoxyglucose, positron emission tomography CT scan identifying tumors that exhibit increased metabolism using glucose, which help to differentiate malignant from benign tumors. (Fig. 1 Im) [20, 22].

The differential diagnosis for cardiac metastases includes thrombi, as exemplified by Case No.4, vegetations, which are the most common causes of cardiac masses [20] and primary cardiac tumors such as myxoma.

Pericardioscopy allows inspection of the periocardium and epicardium and permits tissue acquisition. Pericardioscopy, as a diagnostic tool, is especially powerful in assessment of pericardial disease of unknown origin, [23] particularly when combined with pathological and molecular methods. However, the technique is quite demanding and can be performed only in a limited number of experienced tertiary referral centers.

Cytological evaluation of pericardial effusion can identify malignant cells. About 24% of all cases assessed by pericardial fluid cytology are attributed to malignancy [24]. The leading cause for pericardial effusion is lung adenocarcinoma followed by breast carcinoma in women. Cytology is superior to pericardial biopsy in diagnosing metastatic carcinoma (14.7% false-negative rate for cytology vs., 40% for pericardial biopsy). Other tumors may go undetected in the pericardial fluid. There is no available data on the association of MCTs with pericardial effusion.

Pathological evaluation remains the most definite method for differentiating neoplastic from non-neoplastic cardiac masses [20]. While preoperative diagnostic cardiac biopsies are rarely performed, postoperative pathological evaluation following resection of these tumors, are always performed regardless of their nature.

### Treatment modalities and outcome

Clinical presentation will define the most appropriate management modality. As cardiac metastases are seen in patients with advanced malignant disease, many patients will have already undergone surgical treatment or receive radio/chemotherapy for the original tumor. For the majority of patients, the goal of the treatment is to provide palliation of the symptoms and prevent tumor recurrence.

Cardiac tamponade necessitates immediate pericardiocentesis followed by local administration of chemotherapeutics or radioisotopes to prevent recurrence [15]. If life threatening arrhythmias are detected, they will be the focus of the treatment, including radiofrequent ablation for uncontrollable arrhythmias [25]. In selected cases, chemo embolization of coronary arteries supplying a single cardiac mass may be effective [26].

MCTs present a particular challenge for heart surgeons and need to be treated in specialized cardiac centers that have experience over the whole spectrum of heart surgery. Owing to the small numbers of cases, there are no evidence-based guidelines for the optimal treatment regimen; nevertheless, surgical management should be considered in individual cases. Surgery may be critical and represents the treatment of choice in cases in which prognosis is good, when there are no other metastatic sites, and the tumor can be removed in toto or in cases of poor expected outcome if intra-cardiac obstruction is present [27]. Following, adjuvant radio/chemotherapy should be employed. Because of the multi-modalities of treatment, a multidisciplinary team of cardiologist, oncologist and cardiothoracic surgeon, working with the patient, should contribute to the therapeutic decision-making process. Case No. 1 gives an exceptional example to the effectiveness of multidisciplinary team-work which combines surgery with radio/chemotherapy and cutting edge biological treatments and targeted medications.

## Conclusions

To conclude, the prevalence of malignant diseases continuously rises around the globe. With the improvement of early detection, development of modern diagnostic tools, advances in chemotherapeutic regimens and radiation techniques and refinement of preoperative care, cancer patient survival is expected to increase and therefore the incidence of MCT’s is also expected to rise. Scanning for metastases to the heart is not a routine practice for patients with malignancies and is not currently recommended in any guidelines. We suggest that oncologic patients presenting with cardiopulmonary syndromes would be evaluated for possible cardiac metastases. Multicenter registries, multidisciplinary approach and follow up of the primary tumors will guide more specific and tumor dependent recommendations for the best diagnostic workup. Patients, cardiologists, oncologists and cardiovascular surgeons should be involved in making final decision about the best possible treatment of cardiac metastasis.

## Abbreviations

CT: Computed tomography
MCTs: metastatic cardiac tumors
MRI: Magnetic resonance imaging
SMC: Sheba Medical Center
TICDV: The Institute for Cardiovascular Diseases of Vojvodina
